# Evaluation of whole-body modalities for diagnosis of multifocal osteonecrosis—a pilot study

**DOI:** 10.1186/s13075-021-02473-3

**Published:** 2021-03-11

**Authors:** Shunichi Yokota, Keita Sakamoto, Yukie Shimizu, Tsuyoshi Asano, Daisuke Takahashi, Kohsuke Kudo, Norimasa Iwasaki, Tomohiro Shimizu

**Affiliations:** 1 Department of Orthopaedic Surgery, Faculty of Medicine and Graduate School of Medicine, Hokkaido University, Kita-15 Nishi-7, Kita-ku, Sapporo, 060-8638 Japan; 2 Department of Diagnostic Imaging, Faculty of Medicine and Graduate School of Medicine, Hokkaido University, Kita-15 Nishi-7, Kita-ku, Sapporo, 060-8638 Japan; 3 Department of advanced diagnostic imaging development, Faculty of Medicine and Graduate School of Medicine, Hokkaido University, Kita-15 Nishi-7, Kita-ku, Sapporo, 060-8638 Japan

**Keywords:** Osteonecrosis, Whole-body magnetic resonance imaging, Whole-body bone scintigraphy, Steroid

## Abstract

**Background:**

This study aimed to investigate the ability of whole-body bone scintigraphy (WB-BS) in the detection of multifocal osteonecrosis (ON) compared to whole-body magnetic resonance imaging (WB-MRI) and to clarify the characteristics of patients with multifocal ON among those with ON of the femoral head (ONFH) using WB-MRI.

**Methods:**

Forty-six patients who had symptomatic ONFH and underwent surgery in our hospital from April 2019 to October 2020 were included in the study. Data on patient demographics, including age, sex, body mass index (BMI), history of corticosteroid intake, alcohol abuse, smoking, and symptomatic joints, were collected from their medical records. All patients underwent WB-MRI and WB-BS before surgery.

**Results:**

The agreement in the detection of ON by WB-MRI vs the uptake lesions by WB-BS in the hip joints was moderate (*κ* = 0.584), while that in other joints was low (*κ* < 0.40). Among the 152 joints with ON detected by WB-MRI, 92 joints (60.5%) were symptomatic, and 60 joints (39.5%) were asymptomatic. Twelve out of the 46 (26.0%) patients had multifocal (three or more distinct anatomical sites) ON. Nonetheless, while WB-BS detected symptomatic ON detected by WB-MRI as uptake lesions in 82.6% (76/92) of the joints, asymptomatic ON detected by WB-MRI was detected as uptake lesions in 21.7% (13/60) of the joints. All patients with multifocal ON had a history of steroid therapy, which was significantly higher than that in patients with oligofocal ON (*P* = 0.035). The patients with a hematologic disease had multifocal ON at a higher rate (*P* = 0.015).

**Conclusions:**

It might be difficult for WB-BS to detect the asymptomatic ON detected by WB-MRI compared to symptomatic ON. Considering the cost, examination time, and radiation exposure, WB-MRI might be useful for evaluating multifocal ON. Larger longitudinal studies evaluating the benefits of WB-MRI for detecting the risk factors for multifocal ON are required.

## Background

Osteonecrosis (ON) is characterized by the death of osteocytes due to inadequate blood supply caused by various underlying mechanisms [1]. Although various joints can be affected, including the shoulders, knees, and ankles, ON of the femoral head (ONFH) is most common, with an annual incidence rate of 10,000 to 20,000 new cases in the USA and 2000 to 3000 new cases in Japan, which is expected to increase continuously [2–4]. Multifocal ON is a rare disorder involving three or more distinct anatomical sites and occurs in approximately 3% of all ON patients [5].

Imaging is used to diagnose ON and evaluate the severity of lesions. Radiographs are often used initially, but the early-stage disease is usually undetectable because the radiographic abnormalities develop only after prolonged ischemic changes [6, 7]. A whole-body bone scan (WB-BS) is more sensitive than a simple X-ray scan for the diagnosis of ON [8–10] and can also be used to screen for multifocal ON [7, 11]. However, some reports have suggested that the sensitivity of WB-BS for the diagnosis of symptomatic ON is low [12, 13]. Therefore, the advantages of WB-BS for screening multifocal ON remain debatable.

Magnetic resonance imaging (MRI), on the other hand, is the gold standard for the diagnosis of ON and evaluation of its severity, with high specificity and sensitivity [14, 15]. Conventional MRI includes limited scans and does not cover all joints because of the high cost [7]. Whole-body (WB) MRI (WB-MRI) has emerged in clinical use for the management of inflammatory diseases such as rheumatoid arthritis, spondylarthritis, polymyositis, and dermatomyositis [16, 17]. It also helps in the early detection of osteonecrotic lesions in patients with a hematologic disease treated with corticosteroids [18, 19]. Considering the high accuracy of MRI in the early identification of ON, WB-MRI allows all potential sites of ON to be evaluated in a single examination, thereby improving the prognosis.

However, there is little evidence supporting its utility in the detection of multifocal ON. Moreover, no studies have compared WB-MRI and WB-BS for the detection of ON. Therefore, this study aimed to investigate the ability of WB-BS in the detection of multifocal ON compared to WB-MRI and to clarify the characteristics of patients with multifocal ON among those with ONFH using WB-MRI.

## Methods

### Patients

This cross-sectional study was conducted in accordance with the ethical standards of the Declaration of Helsinki and approved by our institutional review board (# 020-0059). All patients were informed about this study and agreed to its publication. In total, 46 patients who had symptomatic ONFH and underwent surgery in our hospital from April 2019 to October 2020 were eligible to participate in this study. All patients were diagnosed of ONFH by three orthopedic surgeons (TA, DT, and TS) according to the diagnostic criteria reported by Sugano et al. [20]. Additionally, all patients underwent WB-MRI and WB-BS preoperatively.

Data on patient demographics, including age, sex, body mass index (BMI), history of corticosteroid intake, alcohol abuse, and smoking, were collected from their medical records. Alcohol abuse was defined as the consumption of more than 400 mL of alcohol per week, which is known to be a significant risk factor for ONFH [21].

### Whole-body MRI

**Fig. 1:**
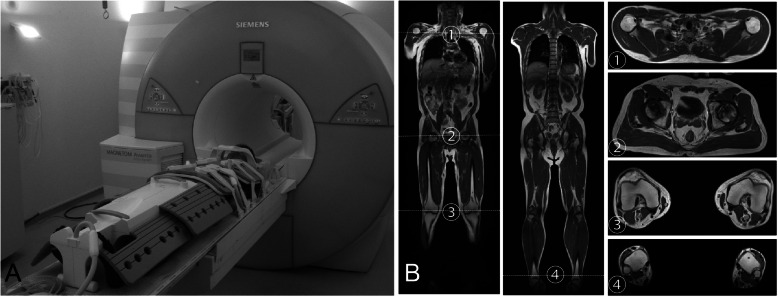
Whole-body MRI. **a** Set-up of coils (head, chest, abdomen, and extremity coils) in a wide bore magnet device. **b** Typical coronal and axial images using T1-weighted (T1W) turbo spin-echo images of whole-body MRI. (1) Shoulder joints, (2) hip joints, (3) knee joints, and (4) ankle joints. MRI, magnetic resonance imaging

WB-MRI was performed using a 1.5-T MRI system (Magnetom Avanto; Siemens Healthcare, Erlangen, Germany) (Fig. 1a). T1-weighted (T1W) turbo spin-echo images (TR 600–800 ms, TE 14 ms, slice thickness 6 mm, slice gap 12 mm in the axial section and with 3 mm in the coronal section) were obtained from the neck to the ankles (Fig. 1b). The total scan time was 20–25 min. While ON is asymptomatic before the collapse and is characterized by a band-like pattern in the T1-weighted images, it becomes symptomatic and painful after the collapse because of bone marrow edema and joint effusion. Therefore, in this study, T1-weighted images were selected because they help in evaluating asymptomatic ON. All MRIs were performed during the hospitalization.

### Whole-body bone scintigraphy

WB-BS was performed 4 h after injection of 555 MBq technetium-99 m hydroxymethylene diphosphonate (Tc-99 m HDP). Anterior and posterior views were acquired using a dual-headed gamma camera system (ECAM, Siemens Healthcare, Erlangen, Germany). Images were analyzed on a Siemens ESOFT Workstation.

### Image interpretation and analysis

Two board-certified radiologists with 12 years of experience (K.S. and Y.S.), blind to the clinical information, evaluated the WB-MRI and WB-BS images. ON was defined as either a subchondral or an intramedullary area demarcated by a distinct marginal rim with a low signal intensity that encompassed the medullary fat on the MRI images. For evaluation of WB-BS, bone regions exhibiting uptake were recorded as previously described [7].

### Statistical analysis

To compare the characteristics of cases with multifocal vs oligofocal ON, continuous and categorical variables were analyzed by independent Student’s *t*-test and Pearson’s chi-square test, respectively*. P*-values < 0.05 were considered significant. Cohen kappa coefficient of the agreement was calculated to evaluate the reproducibility and accuracy of the analysis.

## Results

The demographics and clinical data of the patients are summarized in Table 1. In total, 36 out of the 46 patients received steroid therapy such as prednisolone. Of these 36 patients, 20 had collagen disease, including systemic lupus erythematosus (SLE), dermatomyositis (DM), and polymyositis (PM); 6 had hematologic malignancies; 4 had allergies; 2 had pneumonia; and 4 patients had other diseases. Fifteen out of the 36 patients received steroid pulse therapy, with a mean maximum dose of 50.8 mg (9–90 mg) per day. Nineteen and 28 patients had a history of alcohol abuse and smoking, respectively.

**Table 1:** Patient demographics

46 patients	
Age, years	47.1 (16.0)
Sex, male to female	23:23
Steroid use, cases	36
Comorbidity for steroid use, cases	
Collagen disease	21
Hematologic malignancy	6
Allergy	4
Pneumonia	2
Others	4
Steroid pulse therapy, cases	15
Mean maximum steroid dose, mg	50.8 (17.5)
Alcohol abuse, cases	19
Smoking, cases	28

Data are represented as the mean (standard deviation)

WB-MRI detected osteonecrotic lesions in 82 hip joints of 46 patients, 40 knee joints of 24 patients, 17 ankle joints of 11 patients, 9 shoulder joints of 5 patients, and 4 elbow joints of 3 patients (Table 2). Among the 152 joints evaluated, WB-MRI detected symptomatic and asymptomatic ON in 92 (60.5%) and 60 (39.5%) joints, respectively (Table 3). Twelve out of the 46 patients had multifocal ON detected by MRI. Table 4 compares the demographics of patients with multifocal and oligofocal ON, detected by WB-MRI. All patients with multifocal ON had a history of steroid therapy. Although these patients had a higher ratio of steroid use than those with oligofocal ON (*P* = 0.035), there were no significant differences in the history of steroid pulse therapy and maximum dose of steroid use between the two groups. The patients with hematologic disease had multifocal ON at a higher rate (*P* = 0.015). There were also no significant differences in age, sex, alcohol abuse, and smoking status between the two groups.

**Table 2:** Number of cases of ON detected by WB-MRI and uptake lesions by WB-BS

	ON detected by WB-MRI		Uptake lesions by WB-BS		
	Patient #	Joint #	Patient #	Joint #	*κ* statistic
Hip joint	46	82	46	71	0.584
Knee joint	24	40	16	18	0.069
Ankle joint	11	17	13	21	0.135
Shoulder joint	5	9	16	30	0.244
Elbow joint	3	4	5	7	0.067
Total		152		147	
Multifocal cases	12		14		

*WB-MRI* whole-body magnetic resonance imaging, *WB-BS* whole-body bone scintigraphy, *ON* osteonecrosis, “#” number

**Table 3:** Number of symptomatic and asymptomatic patients with radiological abnormality

	WB-MRI			WB-BS		
	Joint #	Sympt. #	Asympt. #	Joint #	Sympt. #	Asympt. #
Hip joint	82	74	8	71	68	3
Knee joint	40	13	27	18	5	13
Ankle joint	17	2	15	21	2	19
Shoulder joint	9	4	5	30	4	26
Elbow joint	4	0	4	7	0	7
Total	152	92	60	147	76	71

*WB-MRI* whole-body magnetic resonance imaging, *WB-BS* whole-body bone scintigraphy, *Sympt* symptomatic, *Asympt* asymptomatic, “#” number

**Table 4:** Comparison of demographics between patients with multifocal and oligofocal ON

	Multifocal ON (*N* = 12)	Oligofocal ON (*N* = 34)	*P*-value
Age, years	42.6 (12.2)	48.7 (16.9)	0.875
Sex, male to female	4:8	15:19	0.179
Steroid use, cases	12	24	0.034
Steroid pulse therapy, cases	6	9	0.135
Mean maximum steroid dose, mg	58.0 (20.1)	47.5 (15.8)	0.198
Collagen disease, cases	6	15	0.725
Hematologic disease, cases	4	2	0.015
Alcohol abuse, cases	5	14	0.976
Smoking, cases	6	22	0.370

Data are represented as the mean (standard deviation)

*ON* osteonecrosis

**Fig. 2:**
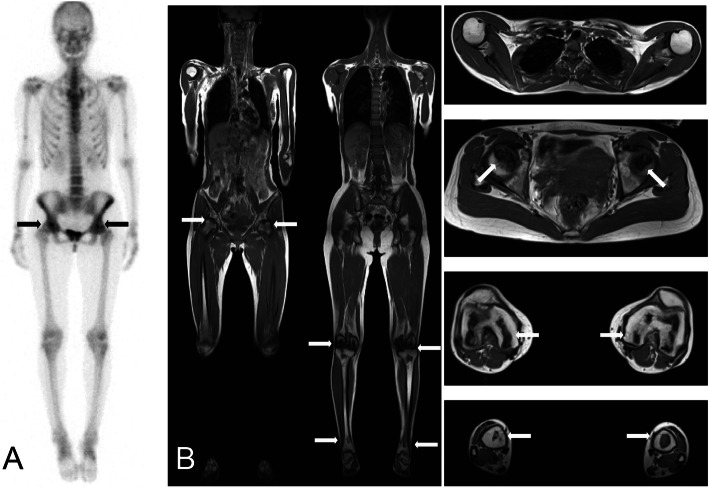
Osteonecrosis of the bilateral hip, knee, and ankle joints in a 38-year-old woman with a history of steroid use for scleroderma. **a** Coronal whole-body bone scintigraphy image. The arrows indicate the region of uptake in the bilateral hip joints. **b** Coronal and axial whole-body magnetic resonance image. The arrows indicate osteonecrotic regions in the bilateral hip, knee, and ankle joints

However, WB-BS detected uptake lesions in 71 hip joints of 46 patients, 18 knee joints of 16 patients, 21 ankle joints of 13 patients, 30 shoulder joints of 16 patients, and 7 elbow joints of 5 patients (Table 2). While the agreement of the detection of ON in the hip joints was moderate and comparable between WB-MRI and WB-BS, that in the knee, ankle, shoulder, and elbow joints showed lower agreement (Table 2) (Fig. 2). WB-BS detected symptomatic uptake lesion in 76/147 (51.7%) joints (Table 3). Although WB-BS detected symptomatic ON detected by WB-MRI as uptake lesions in 82.6% (76/92) of the joints, asymptomatic ON detected by WB-MRI was detected as uptake lesions in 21.7% (13/60) of the joints.

The interobserver variabilities in the detection of ON by WB-MRI and WB-BS between two observers (K.S. and Y.S.) were 0.742 (good) and 0.554 (moderate), respectively. When they had a different diagnosis on the same patients, the radiologists and orthopedic surgeon (TS) discussed and consented if the region was adopted as an occurrence of ON or not.

## Discussion

To clarify if WB-BS uptake lesions are useful for diagnosing the multifocal ON, this study aimed to compare the detection of ON by WB-MRI and WB-BS. The main limitation of this study was that we did not perform a bone biopsy for definitive diagnosis other than femoral head received THA; therefore, this study could not evaluate the sensitivity and specificity of each modality. However, considering that previous studies reported that MRI is the gold standard for the diagnosis of ON and evaluation of its severity, with high specificity and sensitivity [14, 15], we believe that WB-MRI is an attractive and accurate tool to screen the multifocal ON. In this current study, we found that WB-BS could detect symptomatic ON in the lower limbs in more than 80% of the detected cases by WB-MRI, while it was not as effective in detecting asymptomatic ON in the upper limbs. These findings suggest that WB-BS is less efficient in detecting pre-collapse in joints other than the hip joint, where pre-collapse ON has a “cold-in-hot” appearance [20]. Although a previous study showed that pinhole bone scan of the shoulder, hip, knee, and ankle could provide better diagnostic values [22], WB-BS might not be enough resolution for screening multifocal ON, including asymptomatic ON. Recent studies have shown the effectiveness of regenerative therapy using growth factor or cell for ON before a potential collapse [23, 24]. Therefore, early diagnosis of ON might be important in asymptomatic patients. Additionally, considering the examination time (WB-MRI, 20–30 min, and WB-BS, 4 h after injection), cost (WB-MRI, 200 dollars, and WB-BS, 500 dollars, in Japan), and the radiation exposure, indication of WB-BS for screening ON should be considered carefully.

This current study also investigated the clinical characteristics of patients with multiple ON. The incidence of multifocal ON in this study (26.0%) was higher than that reported previously [5, 7]. This discrepancy could be due to the sensitivity of WB-MRI, allowing detection of asymptomatic ON. WB-MRI has been recently reported to detect multifocal ON in 20% (3 of 15) of the patients with polymyositis/dermatomyositis (PM/DM) [19] and in 86% (6 of 7) of the patients with Hodgkin’s lymphoma treated by chemotherapy [25]. Consistent with these reports, this current study showed that the ONFH patients with hematologic disease had multifocal ON with high frequency. The incidence of multifocal ON is high in the presence of clinical risk factors, such as connective tissue disorders including SLE, renal transplantation, leukemia, HIV, coagulation disorders, and sickle cell disease [7, 26, 27]. Because this current study included patients with various comorbidities and symptomatic ONFH, future studies should investigate the incidence of multifocal ON among the patients with each comorbidity.

Consistent with the previous reports [28], this current study showed that multifocal ON occurs in steroid associated cases of ONFH. Contrary to a report by Zhang et al. [29], we found no significant difference in the maximum steroid dose for steroid pulse therapy between patients with multifocal and oligofocal ON. This discrepancy could be because Zhang et al. studied patients with severe acute respiratory syndrome [29]. Therefore, future studies should also investigate the association between the incidence of multifocal ON and the associated risk factors such as steroid usage under the condition of unified cases.

The present study had several limitations. First is the small sample size, which included patients with multifocal ON and various other comorbidities. Larger and longitudinal cohort studies using WB-MRI should be conducted to detect the risk factors for multifocal ON. Second, this study lacked bone biopsy and pathological data. There is a possibility that other diseases such as subchondral insufficiency fracture or rapidly destructive coxarthrosis were misdiagnosed as ON. Third, the study has been conducted only on patients with symptomatic ONFH admitted to the hospital for surgical procedures. Hence, there is a selection bias as well.

## Conclusions

In conclusion, it might be difficult for WB-BS to detect the asymptomatic ON detected by WB-MRI compared to symptomatic ON. Considering the cost, examination time, and radiation exposure, WB-MRI might be useful for evaluating multifocal ON. Larger longitudinal studies evaluating the benefits of WB-MRI for detecting the risk factors for multifocal ON are required.

## Data Availability

The datasets used and/or analyzed during the present study are available from the corresponding author on reasonable request.

## Abbreviations

BMI: Body mass index
MRI: Magnetic resonance imaging
ON: Osteonecrosis
ONFH: Osteonecrosis of femoral head
T1W: T1-weighted
WB-BS: Whole-body bone scintigraphy
WB-MRI: Whole-body magnetic resonance imaging
